# Supplementary material for: Dynamic transcriptomic profiles of zebrafish gills in response to zinc supplementation
Source: BMC Genomics. 2010 Oct 11;11:553. doi: 10.1186/1471-2164-11-553 (PMC3091702; doi:10.1186/1471-2164-11-553)
Supplement: Additional file 2 — Interactive Direct Interaction Network representing the molecular interactions between zinc, copper, iron, calcium and proteins encoded by transcripts changed by zinc supplementation. Mini web-site containing index.html and hyperlinked pages in subdirectory describing a Direct Interaction Network automatically generated based on curated interactions contained within the proprietary PathwayArchitect database. Ovals represent proteins and the circles symbolize metal ions. Objects are coloured by their abundance in zebrafish at the time-point they were significantly different from the control is a scale from -4 fold (dark green) to +4 fold (dark red). Where significant differences were found at more than one time-point, the colour overlay shows expression at the first instance. Dark blue squares denote 'binding', and light blue squares 'expression'; green squares stand for 'regulation', green diamonds for 'metabolism', and green circles for 'promoter binding'. Arrow heads indicate directionality of the interaction where annotated. All nodes and edges can be further interrogated by selecting the relative area of the image. [file 1471-2164-11-553-S2.zip › PathwayArchitect Zn xs DIN/158929.html]

# PROTEIN: FOXM1

|  |  |
| --- | --- |
| Name | FOXM1 |
| Type | PROTEIN |
| Description | forkhead box M1 |
| Note | Putative transcription factor; may play a role in the control of cell proliferation [RGD] |
| Alias | Forkhead homolog 16 |
|  | forkhead (Drosophila)-like 16 |
|  | Winged-helix transcription factor Trident |
|  | Forkhead, drosophila, homolog-like 16 |
|  | Winged helix factor from INS-1 cells |
|  | HFH-11 |
|  | HFH-11B |
|  | FKHL16 |
|  | Win |
|  | INS-1 |
|  | HFH11 |
|  | MPP-2 |
|  | Mpm2 |
|  | MPP2 |
|  | Fkh16 |
|  | INS-1 winged helix |
|  | forkhead homolog 16 (Drosophila) |
|  | TRIDENT |
|  | Foxm1 |
|  | AW554517 |
|  | WIN |
|  | Trident |
|  | Foxm1b |
|  | HNF-3 |
|  | FOXM1B |
|  | MPHOSPH2 |


---

|  |  |
| --- | --- |
| GO Component | transcription factor complex |
|  | nucleus |


---

|  |  |
| --- | --- |
| GO ID | GO:0005634 |
|  | GO:0003677 |
|  | GO:0001889 |
|  | GO:0006355 |
|  | GO:0042127 |
|  | GO:0003702 |
|  | GO:0006366 |
|  | GO:0006979 |
|  | GO:0005667 |
|  | GO:0006350 |
|  | GO:0003700 |


---

|  |  |
| --- | --- |
| MIM | MIM:602341 |


---

|  |  |
| --- | --- |
| Connectivity | 392 |


---

|  |  |
| --- | --- |
| Entrez ID | 2305 |
|  | 58921 |
|  | 14235 |


---

|  |  |
| --- | --- |
| Agilent ID | A\_53\_P147494 |
|  | A\_51\_P245103 |
|  | A\_52\_P28806 |
|  | A\_23\_P151150 |
|  | A\_53\_P179040 |
|  | A\_42\_P527141 |
|  | A\_14\_P120375 |


---

|  |  |
| --- | --- |
| Cellular Localization | Nucleus |
|  | Organelle |
|  | Cell |


---

|  |  |
| --- | --- |
| Pathway | Hedgehog Signaling |
|  | Zn xs inventory |
|  | Zn xs DIN |


---

|  |  |
| --- | --- |
| GO Process | response to oxidative stress |
|  | regulation of transcription, DNA-dependent |
|  | liver development |
|  | transcription |
|  | regulation of cell proliferation |
|  | transcription from RNA polymerase II promoter |


---

|  |  |
| --- | --- |
| UniGene | Hs.239 |
|  | Mm.42148 |
|  | Rn.10665 |


---

|  |  |
| --- | --- |
| Affymetrix Probeset ID | 107523\_at |
|  | 1369018\_at |
|  | 1417748\_x\_at |
|  | 1437138\_at |
|  | 1448833\_at |
|  | 1448834\_at |
|  | 202580\_x\_at |
|  | 1453107\_s\_at |
|  | 34715\_at |
|  | 1392058\_at |
|  | 70225\_r\_at |
|  | 98305\_at |
|  | 98306\_g\_at |
|  | g11386144\_3p\_s\_at |
|  | U74612\_at |
|  | U83112\_at |
|  | 214148\_at |
|  | 41323\_at |
|  | 41324\_g\_at |
|  | Hs.239.3.A1\_3p\_at |
|  | TC37895\_at |
|  | rc\_AA944677\_at |


---

|  |  |
| --- | --- |
| GO Function | RNA polymerase II transcription factor activity |
|  | transcription factor activity |
|  | DNA binding |


---

|  |  |
| --- | --- |
| Nucleotide | AK041638 |
|  | AK187908 |
|  | DQ022289 |
|  | NM\_202002 |
|  | U83112 |
|  | NM\_021953 |
|  | BC006788 |
|  | BC075723 |
|  | NM\_008021 |
|  | BQ229497 |
|  | NM\_202003 |
|  | BC035437 |
|  | AK182102 |
|  | U74613 |
|  | AK213171 |
|  | U74612 |
|  | BC012863 |
|  | U83113 |
|  | BT006986 |
|  | NM\_031633 |
|  | BC006192 |
|  | BC006529 |
|  | AK189398 |
|  | BC065067 |
|  | L16783 |
|  | AK049177 |
|  | Y11245 |


---

|  |  |
| --- | --- |
| Protein | AAH06192 |
|  | NP\_068772 |
|  | Q08050 |
|  | NP\_032047 |
|  | AAC51128 |
|  | NP\_973731 |
|  | AAH65067 |
|  | AAH06529 |
|  | P97691 |
|  | AAC37541 |
|  | AAC51129 |
|  | NP\_113821 |
|  | NP\_973732 |
|  | CAA72115 |
|  | AAH75723 |
|  | BAC33588 |
|  | O08696 |
|  | AAH12863 |
|  | AAP35632 |
|  | AAY26401 |
|  | AAH06788 |
|  | AAC63595 |
|  | AAC63594 |


---

|  |  |
| --- | --- |
| Organism | Mammal |


---

|  |  |
| --- | --- |
| Location | chromosome 12, 12p13 (Homo sapiens) |
|  | chromosome 6, 6 62.0 cM, 6 F3 (Mus musculus) |
|  | 6 62.0 cM (Mus musculus) |
|  | chromosome 4, 4q42 (Rattus norvegicus) |


---

|  |  |
| --- | --- |
